# Postoperative D-dimer–based Screening for Venous Thromboembolism after Total Hip Arthroplasty: A Retrospective Cohort Study of 1,651 Hips

**DOI:** 10.31662/jmaj.2026-0205

**Published:** 2026-07-31

**Authors:** Hironori Kojima, Hisatoshi Ishikura, Masashi Sato, Mayu Iiboshi, Masaki Hatano, Takeyuki Tanaka, Sakae Tanaka

**Affiliations:** 1 Department of Orthopaedic Surgery, The University of Tokyo, Tokyo, Japan; 2 Department of Orthopaedic Surgery, NTT Medical Center Tokyo, Tokyo, Japan

**Keywords:** total hip arthroplasty, D-dimer, venous thromboembolism, deep vein thrombosis, pulmonary embolism, contrast-enhanced computed tomography

## Abstract

**Introduction:**

Venous thromboembolism (VTE), including deep vein thrombosis (DVT) and pulmonary embolism (PE), is a serious complication after total hip arthroplasty (THA). Although D-dimer testing is widely used for VTE screening, there is no consensus regarding its optimal cutoff value or screening strategy after THA. The purpose of this study was to evaluate the validity of a postoperative VTE screening strategy based on a D-dimer cutoff value of 10.0 μg/mL measured at 1 week after unilateral THA.

**Methods:**

This retrospective observational study included 1,651 unilateral THAs at a single institution between April 2007 and March 2023. Serum D-dimer levels were routinely measured at 1 week postoperatively. Patients with D-dimer levels ≥10.0 μg/mL underwent contrast-enhanced computed tomography (CT) to evaluate for DVT and/or PE. Demographic, clinical, and perioperative variables were analyzed, and multivariable regression analyses were performed to identify factors associated with postoperative D-dimer levels and VTE.

**Results:**

Postoperative D-dimer levels ≥10.0 μg/mL were observed in 343 hips (20.8%), among which 31 hips were diagnosed with VTE. Of these, 22 hips had isolated DVT, seven had isolated PE, and two had both DVT and PE. Appropriate anticoagulation therapy was initiated in all diagnosed cases, and no fatal or clinically severe VTE events occurred. Patients with VTE had significantly higher D-dimer levels compared to those without VTE. Multivariable analysis demonstrated that female sex, advanced age, diabetes treated with medication, and greater intraoperative blood loss were independently associated with higher postoperative D-dimer levels.

**Conclusions:**

A postoperative D-dimer–based screening strategy incorporating contrast-enhanced CT for patients with D-dimer levels ≥10.0 μg/mL at 1 week after THA appeared to be feasible and clinically acceptable, with no severe thromboembolic events observed in this cohort of 1,651 hips. However, further studies are warranted to determine the optimal cutoff value for postoperative D-dimer screening.

## Introduction

Total hip arthroplasty (THA) is a widely performed surgical intervention for alleviating pain and restoring function in patients with advanced hip osteoarthritis, thereby facilitating their return to daily activities and social reintegration ^(1), (2)^. However, venous thromboembolism (VTE), including deep vein thrombosis (DVT) and pulmonary embolism (PE), remains one of the most serious complications following THA. According to the American College of Chest Physicians guidelines, patients undergoing THA are at particularly high risk for both asymptomatic DVT (40%-60%) and symptomatic VTE (2%-5%), and in the absence of thromboprophylaxis, fatal PE may occur in approximately 1 in 300 patients ^(3)^. In contemporary practice, symptomatic PE has been reported to occur in approximately 0.2% to 1% of patients after total joint arthroplasty, with fatal PE occurring in approximately 0.1% to 0.3% of cases despite modern thromboprophylaxis ^(4), (5)^. Once VTE develops, the risk of mortality among affected patients has been reported to be as high as several percent ^(6)^. In particular, proximal DVT carries a substantial risk of PE and post-thrombotic syndrome ^(3), (6)^. These findings underscore the importance of identifying VTE prior to the onset of fatal PE and ensuring timely initiation of appropriate treatment when present. Despite advances in perioperative management and routine thromboprophylaxis, postoperative VTE continues to pose an important clinical concern after THA.

D-dimer, a fibrin degradation product, is widely used as a screening biomarker for VTE because of its high sensitivity but low specificity ^(3)^. Although low D-dimer levels are useful for excluding acute thrombosis, elevated levels are frequently observed after major orthopedic surgery owing to surgical trauma and postoperative inflammation, limiting its specificity in the postoperative setting ^(3)^. Consequently, there is no consensus regarding the optimal timing or cutoff value of D-dimer measurement for VTE screening after THA, and institutional screening strategies vary widely.

Previous orthopedic studies have suggested that a D-dimer cutoff value of 10.0 μg/mL measured on postoperative day 7 provides high sensitivity and specificity for the diagnosis of DVT when venography is used as the reference standard ^(4)^. However, other studies have proposed different thresholds and timing for postoperative D-dimer assessment. For example, ultrasonographic screening has been performed in patients with D-dimer levels ≥20.0 μg/mL on postoperative days 7, 14, and 21 after THA ^(7)^. In contrast, a lower cutoff value of ≥5 μg/mL on postoperative day 3 has been reported to be useful for DVT detection in gynecologic cancer surgery ^(8)^, while in gastrointestinal surgery, serial changes in D-dimer levels across postoperative days 1, 3, 5, and 7 have been suggested to improve risk prediction ^(9)^. Taken together, there is no clear consensus regarding the optimal postoperative D-dimer cutoff value or the appropriate imaging modality to be used when the threshold is exceeded. Furthermore, our institutional protocol—performing contrast-enhanced computed tomography (CT) in patients with D-dimer levels ≥10.0 μg/mL—has not been sufficiently evaluated in large clinical cohorts, underscoring the importance of accumulating further evidence.

Therefore, the purpose of this retrospective study was to evaluate the validity of a postoperative VTE screening strategy based on a D-dimer cutoff value of 10.0 μg/mL measured at 1 week after unilateral THA, followed by selective contrast-enhanced CT, and to identify factors associated with postoperative D-dimer elevation. We hypothesized that a cutoff of 10.0 μg/mL would identify patients at increased risk of postoperative VTE.

## Materials and Methods

### Patients

This retrospective observational study was conducted at a single tertiary referral center. The study protocol was approved by the institutional review board of our institution (approval number 2674-10), and written informed consent for the use of perioperative data and postoperative outcomes for research purposes was obtained from all patients.

We reviewed consecutive patients who underwent unilateral THA at our institution between April 2007 and March 2023. To reduce selection bias, consecutive eligible patients were included. To minimize measurement bias, postoperative D-dimer was routinely measured at a fixed time point (postoperative day 7) according to the institutional protocol. The study size was determined by the number of consecutive eligible patients available during the study period.

Patients were excluded if they met any of the following criteria:

1. History of previous hip surgery;

2. THA performed for diagnoses other than osteoarthritis, including osteonecrosis, traumatic conditions, or inflammatory arthritis;

3. Occurrence of perioperative complications, such as fracture or infection, that resulted in a substantial deviation from the standard postoperative rehabilitation protocol;

4. Allergy to contrast agents;

5. Inability to complete a minimum follow-up period of 2 years.

All procedures were performed using a posterior approach in the lateral decubitus position. Postoperative rehabilitation was initiated on the first postoperative day, and patients were encouraged to begin ambulation with weight bearing as tolerated. For pharmacological prophylaxis, since edoxaban became available in Japan in 2011, our institutional protocol has consisted of administering edoxaban 15 mg/day for 2 weeks postoperatively. Prior to the introduction of edoxaban, fondaparinux 1.5 mg/day was administered subcutaneously for 2 weeks after surgery according to the institutional protocol. In patients with allergies or contraindications to the protocol-based medication, alternative agents were used. Furthermore, patients who had been receiving anticoagulant or antiplatelet therapy generally continued the same medication after surgery. These medications included aspirin and warfarin, among others.

### Postoperative VTE screening protocol

At our institution, postoperative screening for VTE following unilateral THA has been standardized. Serum D-dimer levels are routinely measured on postoperative day 7. Patients with D-dimer levels ≥10.0 μg/mL underwent further evaluation with contrast-enhanced CT to assess for the presence of DVT and/or PE. Patients with D-dimer levels below this threshold did not routinely undergo imaging unless clinically indicated.

### Definition of VTE

Postoperative VTE was defined as the presence of DVT and/or PE detected by contrast-enhanced CT performed in patients with elevated D-dimer levels (≥10.0 μg/mL). Both symptomatic and asymptomatic VTE detected by CT were included in the diagnosis of VTE.

Patients with D-dimer levels <10.0 μg/mL were not routinely evaluated with imaging studies, and therefore VTE in these patients could not be assessed.

### Data collection

Demographic and clinical variables included sex, age, body mass index (BMI), diabetes treated with medication, American Society of Anesthesiologists Physical Status (ASA-PS) classification, intraoperative blood loss adjusted for body weight, use of edoxaban, postoperative D-dimer levels, and the presence or absence of postoperative VTE.

### Statistical analyses

Statistical analyses were performed using BellCurve for Excel (Social Survey Research Information Co., Ltd., Tokyo, Japan). Continuous variables were compared using Welch’s t test, and categorical variables were analyzed using the chi-square test or Fisher’s exact test, as appropriate. Multivariable linear regression analysis was performed with postoperative D-dimer level as the dependent variable. Sex, age, BMI, diabetes treated with medication, ASA-PS classification, intraoperative blood loss adjusted for body weight, and use of edoxaban were entered simultaneously into the model based on clinical relevance. The variance inflation factor (VIF) was used to check for multicollinearity in the model, and in accordance with previous reports, a VIF >10 was considered indicative of multicollinearity ^(10)^. A p-value <0.05 was considered statistically significant.

## Results

A total of 1,651 hips were included in this study (Figure 1). The mean D-dimer level at 1 week postoperatively was 7.7 μg/mL. When patients were stratified according to postoperative D-dimer levels, 343 hips (20.8%) were classified into the high D-dimer group (≥10 μg/mL), whereas 1,308 hips (79.2%) were classified into the low D-dimer group (<10 μg/mL). Comparisons between the two groups are shown in Table 1. Patients in the high D-dimer group were significantly older, more frequently female, and had a higher prevalence of diabetes treated with medication. Intraoperative blood loss adjusted for body weight was also significantly greater in the high D-dimer group.

**Figure 1.**
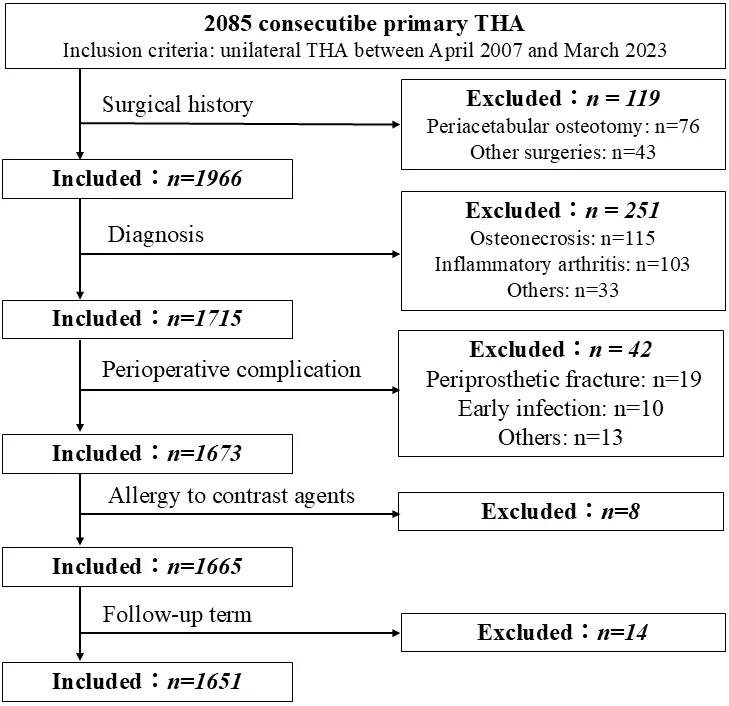
Flow diagram of patient selection. Flow diagram illustrating patient selection for this retrospective study.

**Table 1.** Comparison of Baseline Characteristics between Patients with Postoperative D-Dimer Levels ≥10.0 and <10.0 μg/mL.

Characteristics	D-dimer levels ≥10.0 μg/mL (n = 343)	D-dimer levels <10.0 μg/mL (n = 1,308)	p-Value
Sex: female, n (%)	310 (90.4)	1,068 (81.7)	<0.001*
Age (years), mean	66.1	63.3	<0.001**
BMI (kg/m^2^), mean	23.1	23.3	0.32**
Diabetes treatment, n (%)	48 (14.0)	77 (5.9)	<0.001*
ASA-PS ≥3, n (%)	16 (4.7)	71 (5.4)	0.57*
Intraoperative blood loss (g/body weight 50 kg), mean	441.0	367.0	<0.001**
Postoperative antithrombotic therapy, n (%)			
Edoxaban	223 (65.0)	962 (73.5)	0.002*
Fondaparinux	50 (14.6)	125 (9.6)	0.007*
Other anticoagulant/antiplatelet agents	70 (20.4)	221 (16.9)	0.129*

*Chi-square test; **Welch’s t test. ASA-PS: American Society of Anesthesiologists Physical Status; BMI: body mass index.

Among patients with D-dimer levels ≥10 μg/mL, VTE was detected in 31 hips. Of these, 22 patients had DVT alone, seven had PE alone, and two had both DVT and PE. Among the nine patients with PE, postoperative D-dimer levels ranged from 10.2 to 45.0 μg/mL, with a mean value of 17.2 μg/mL and a median value of 12.7 μg/mL. With respect to anatomical extent, one patient had subsegmental PE, three had segmental PE, and five had lobar PE. Following diagnosis, treatment and follow-up imaging examinations were conducted under the supervision of vascular specialists according to the clinical course of each patient. Among the five patients with lobar PE, one was switched to rivaroxaban, and complete resolution was confirmed by contrast-enhanced CT 1 week later. One patient received an increased dose of edoxaban (60 mg/day), and regression of the embolus was confirmed 1 week later. Three patients underwent heparinization; among them, the patient who underwent follow-up CT at 1 week showed partial resolution, whereas the patients who underwent follow-up CT at 1 month and 3 months showed complete resolution.

Among the 22 patients with isolated DVT, all underwent intensified anticoagulation therapy. Six patients completed treatment without repeat imaging because they remained asymptomatic and showed no clinical signs of DVT. Follow-up CT performed in eight patients demonstrated complete or partial thrombus resolution in seven patients. Follow-up ultrasonography was performed in eight patients and demonstrated improvement in five patients. Among the three patients without apparent improvement on follow-up imaging, two had preoperatively documented DVT and were considered likely to have organized chronic thrombi. No patients developed severe symptoms related to DVT or PE.

Among patients with postoperative D-dimer levels ≥10.0 μg/mL, univariate comparisons between those with and without postoperative VTE are presented in Table 2. Patients who developed VTE had significantly higher postoperative D-dimer levels and were less likely to have received edoxaban. Multivariable linear regression analysis revealed that female sex, advanced age, diabetes treated with medication, and greater intraoperative blood loss were independently associated with higher postoperative D-dimer levels (Table 3).

**Table 2.** Univariate Comparison between Patients with and without VTE among Those with D-Dimer levels ≥10.0 μg/mL.

Variables	VTE(+) (n = 31)	VTE(−) (n = 312)	p-Value
Sex: female, n (%)	29 (93.5)	281 (90.1)	0.53*
Age (years), mean	67.8	66.0	0.33**
BMI (kg/m^2^), mean	23.5	23.0	0.66**
Diabetes treatment, n (%)	3 (9.7)	45 (14.4)	0.47*
ASA-PS ≥3, n (%)	2 (6.5)	14 (4.5)	0.62*
Intraoperative blood loss (g/body weight 50 kg), mean	486.0	436.0	0.38**
No edoxaban use, n (%)	12 (38.7)	58 (18.6)	0.008*
D-dimer level at 1 week (μg /mL), mean	17.1	13.0	<0.001**

*Chi-square test; **Welch’s t test. ASA-PS: American Society of Anesthesiologists Physical Status; BMI: body mass index; VTE: venous thromboembolism.

**Table 3.** Multiple Regression Analysis with D-Dimer Level as the Dependent Variable.

Variables	Partial regression coefficient (95% CI)	p-Value	VIF
Sex: female	1.137 (0.593 to 1.681)	<0.001*	1.02
Age (years)	0.051 (0.036 to 0.072)	<0.001*	1.04
BMI (kg/m^2^)	0.012 (−0.027 to 0.080)	0.62	1.06
Diabetes treatment	1.567 (0.778 to 2.356)	<0.001*	1.05
ASA-PS ≥3	−0.191 (−1.125 to 0.743)	0.63	1.03
Intraoperative blood loss (g/body weight 50 kg)	0.002 (0.002 to 0.003)	<0.001*	1.07
No edoxaban use	0.430 (0.029 to 0.888)	0.07	1.09

*p <0.05. ASA-PS: American Society of Anesthesiologists Physical Status; BMI: body mass index; CI: confidence interval; VIF: variance inflation factor.

## Discussion

VTE remains a clinically important complication after THA ^(11)^. According to the Japanese Orthopaedic Association guideline, the incidence of VTE after THA has been reported to be 10%-30% for DVT and 0.5%-1% for symptomatic PE, with fatal PE occurring in <0.5% of cases ^(12)^. Similarly, previous studies from Western populations have reported that fatal PE after elective THA occurs in approximately 0.08% to 0.12% of cases, despite the use of modern thromboprophylaxis strategies ^(13), (14)^. These findings highlight that although the incidence of fatal PE is relatively low, it remains a nonnegligible and potentially preventable cause of mortality following THA.

D-dimer is widely used in clinical practice as a convenient and relatively low-cost screening tool for VTE ^(15)^. Nevertheless, there remains no consensus regarding the optimal cutoff value or the appropriate timing of measurement in the postoperative setting. Furthermore, these parameters may vary depending on surgical factors, such as the type of procedure and its degree of invasiveness ^(16)^. Given this variability, the accumulation of evidence across diverse institutions and surgical settings is essential to establish a more robust and generalizable screening strategy for postoperative VTE.

Several diagnostic approaches are available to evaluate the presence of VTE in patients identified through D-dimer–based screening. Lower extremity ultrasonography is widely used for the detection of DVT after THA, given its noninvasive nature and feasibility as a bedside examination ^(17)^. However, it has limited ability to evaluate pelvic veins and cannot assess PE. In this study, among the nine cases in which PE was identified, seven were not accompanied by detectable DVT. Considering that the primary objective of VTE screening is to prevent fatal PE, performing contrast-enhanced CT in patients who are flagged by D-dimer screening appears to be a reasonable approach, as it allows for the direct detection of PE and facilitates timely therapeutic intervention.

In our screening protocol, patients with elevated D-dimer levels underwent contrast-enhanced CT at 1 week postoperatively, allowing identification of not only DVT but also PE at an early stage. Reevaluation of contrast-enhanced CT findings demonstrated that more than half of the PE-positive patients had lobar PE, suggesting that the detected emboli were not limited to small peripheral lesions. Following diagnosis, treatment was initiated under the supervision of vascular specialists, typically consisting of escalation of anticoagulation therapy from a prophylactic to a therapeutic dose of edoxaban, with extension of the treatment duration as appropriate. This systematic approach may have contributed to thrombus regression or resolution, as confirmed by follow-up imaging in many patients with PE, and may consequently have contributed to the prevention of severe clinical outcomes in patients with postoperative VTE.

However, this strategy also resulted in approximately 20% of patients undergoing contrast-enhanced CT, raising important considerations regarding its safety and cost-effectiveness. Although contrast-enhanced CT is a reliable modality for detecting PE and proximal DVT, it is not without risks. The use of iodinated contrast agent has been associated with adverse reactions, including hypersensitivity reactions and contrast-induced nephropathy, as summarized in the European Society of Urogenital Radiology guidelines ^(18)^. In addition, previous reports have demonstrated that although most adverse reactions are mild, severe reactions such as anaphylaxis, albeit rare, can occur and may be life threatening ^(19)^. Radiation exposure is another concern, particularly when imaging is applied to a large proportion of postoperative patients. Therefore, while the present screening strategy enabled early detection of VTE and timely therapeutic intervention, its broader application should be carefully evaluated in terms of risk–benefit balance.

With respect to risk factors, patients with VTE identified on contrast-enhanced CT had significantly higher postoperative D-dimer levels than those without VTE. In addition, in the overall cohort, advanced age, female sex, diabetes treated with medication, and greater intraoperative blood loss were associated with elevated postoperative D-dimer levels. Although this study did not demonstrate that these factors were directly associated with the occurrence of DVT or PE—likely due to the limited number of VTE events—previous studies have consistently identified these factors as risk factors for postoperative VTE after THA ^(3), (20), (21), (22), (23), (24), (25)^. Therefore, these patient-related factors should be carefully considered when evaluating postoperative VTE risk.

Among patients with postoperative D-dimer levels ≥10.0 μg/mL, univariate analysis demonstrated that those with VTE had significantly higher D-dimer levels at 1 week after surgery than those without VTE. This finding supports the clinical validity of postoperative D-dimer as a screening biomarker for identifying patients at increased risk of VTE after THA. In addition, among patients with D-dimer levels ≥10.0 μg/mL, the proportion of patients receiving postoperative edoxaban prophylaxis was significantly lower in the VTE-positive group than in the VTE-negative group. This result may reflect the preventive efficacy of edoxaban against postoperative thrombus formation after THA.

Several limitations should be acknowledged. First, this was a retrospective single-center study, and some patients underwent staged bilateral THA, which may have introduced within-patient correlation. Second, postoperative VTE was evaluated only in patients with D-dimer levels ≥10.0 μg/mL, and D-dimer levels were measured only once at 1 week postoperatively without preoperative assessment. Consequently, asymptomatic VTE in patients with lower D-dimer levels and preexisting thrombosis before surgery could not be evaluated. Furthermore, follow-up imaging after VTE diagnosis was performed according to the clinical judgment of vascular specialists. Although available follow-up imaging frequently demonstrated thrombus regression or resolution after treatment, the timing and modality of follow-up examinations were not standardized. Therefore, formal thrombus resolution rates could not be calculated. In addition, although edoxaban was mainly used for VTE prophylaxis in this study, other agents such as aspirin have also been reported to be effective and safe ^(26), (27)^. Therefore, further studies evaluating D-dimer–based screening strategies in patients receiving alternative prophylactic regimens are warranted. Finally, the optimal D-dimer cutoff value for postoperative VTE screening remains uncertain. Although higher D-dimer thresholds may improve the cost-effectiveness of screening by reducing the number of patients undergoing contrast-enhanced CT, caution is warranted because eight of the nine PE-positive patients had D-dimer levels <20.0 μg/mL, and more than half of these patients had lobar PE requiring intensified anticoagulation therapy. Conversely, previous studies have demonstrated that postoperative VTE may occur even in patients with relatively low D-dimer levels ^(28)^, suggesting that some asymptomatic VTE events may have remained undetected among patients with D-dimer levels <10.0 μg/mL in this study. Therefore, further studies are warranted to determine the most appropriate D-dimer cutoff value for postoperative VTE screening after THA.

Despite these limitations, several important clinical implications can be drawn from this study. First, no fatal PE occurred among the entire cohort of 1,651 hips, including patients with postoperative D-dimer levels <10.0 μg/mL. This finding suggests that clinically significant PE was unlikely to have been missed in patients with D-dimer levels below this threshold. Second, among patients with D-dimer levels ≥10.0 μg/mL, contrast-enhanced CT enabled the detection of VTE at an early stage, and appropriate treatment was subsequently initiated. Given that previous studies have reported a measurable incidence of fatal PE after THA ^(11), (12), (13)^, the absence of fatal events in our cohort suggests that this screening and management strategy may represent a clinically useful approach for postoperative VTE surveillance.

In conclusion, the use of a D-dimer cutoff value of 10.0 μg/mL followed by selective contrast-enhanced CT may represent a clinically useful screening strategy after THA. However, further studies are required to determine the optimal D-dimer threshold that balances early detection of clinically significant VTE with the avoidance of unnecessary investigations.

## Article Information

### Author Contributions

Hironori Kojima: conceptualization, data curation, formal analysis, investigation, methodology, and writing – original draft; Hisatoshi Ishikura: conceptualization, methodology, supervision, validation, writing – review and editing, and correspondence; Masashi Sato: data curation, investigation, and writing – review and editing; Mayu Iiboshi: data curation and investigation; Masaki Hatano: formal analysis and writing – review and editing; Takeyuki Tanaka: data curation and investigation; and Sakae Tanaka: supervision, project administration, and writing – review and editing. All authors read and approved the final manuscript.

### Conflicts of Interest

None

### Ethics Approval

The study protocol was approved by the Institutional Review Boards of The University of Tokyo (approval number 2674-10; May 2, 2023). Written informed consent for the use of perioperative data and postoperative outcomes for research purposes was obtained from all patients.

### Disclaimer

Author Sakae Tanaka is one of the Editors of JMA Journal and on the journal’s Editorial Staff. He was not involved in the editorial evaluation or decision to accept this article for publication at all.
